# Herpes Simplex Virus 1 Targets the Murine Olfactory Neuroepithelium for Host Entry

**DOI:** 10.1128/JVI.01748-13

**Published:** 2013-10

**Authors:** Maitreyi Shivkumar, Ricardo Milho, Janet S. May, Michael P. Nicoll, Stacey Efstathiou, Philip G. Stevenson

**Affiliations:** Division of Virology, Department of Pathology, University of Cambridge, Addenbrookes Hospital, Cambridge, United Kingdom

## Abstract

Herpes simplex virus 1 (HSV-1) is a ubiquitous and important human pathogen. It is known to persist in trigeminal ganglia (TG), but how it reaches this site has been difficult to determine, as viral transmission is sporadic, pathogenesis is complicated, and early infection is largely asymptomatic. We used mice to compare the most likely natural HSV-1 host entry routes: oral and nasal. Intranasal infection was 100-fold more efficient than oral and targeted predominantly the olfactory neuroepithelium. Live imaging of HSV-1-expressed luciferase showed infection progressing from the nose to the TG and then reemerging in the facial skin. The brain remained largely luciferase negative throughout. Infected cell tagging by viral Cre recombinase expression in floxed reporter gene mice showed nasal virus routinely reaching the TG and only rarely reaching the olfactory bulbs. Thus, HSV-1 spread from the olfactory neuroepithelium to the TG and reemerged peripherally without causing significant neurological disease. This recapitulation of typical clinical infection suggests that HSV-1 might sometimes also enter humans via the respiratory tract.

## INTRODUCTION

Herpes simplex virus 1 (HSV-1) persists in at least 70% of humans. Infection is mostly mild but can cause repeated ulceration, keratitis, and encephalitis (1). Better control would therefore greatly benefit human health. Established, latent infections are difficult if not impossible to clear, so host entry offers perhaps the best opportunity to break the viral infection cycle. Understanding it is consequently an important goal.

Epidemiology tells us that HSV-1 transmission requires close contact. Sexual contact is one route (2), but virions more usually transfer from carriers to new hosts via saliva. Viral genomes then reach trigeminal ganglia (TG) sensory neurons (3, 4), where they persist in latent form and intermittently reactivate to shed new virions into saliva for further transmission (5). Latent infection is sometimes also found in other sensory ganglia (6, 7) that receive neuronal projections from the same peripheral sites. HSV-1 infection often presents with acute oral (p.o.) and perioral lesions. These could reflect viral replication at an entry site. However, they could also reflect a first wave of virus reemergence from the TG, so-called zosteriform spread (8, 9). Other alphaherpesviruses clearly present with disseminated secondary rather than primary lesions: varicella-zoster virus with the cutaneous rash of chickenpox, Marek's disease virus with shedding from feather follicles (10), and pseudorabies virus with salivary shedding after respiratory infection (11). Thus, clinical description can identify host exit, but host entry, which may follow different routes, must be defined functionally.

Most natural HSV-1 transmission is sporadic and asymptomatic (5). Thus, functional data must come largely from animal models. Nonhumans are unnatural hosts for HSV-1, and xenogeneic infections must be interpreted with caution. However, HSV-1 readily infects murine cells *in vitro*, and mice provide a widely used *in vivo* infection model with proven utility in developing antiviral therapies (12, 13). HSV-1 can infect both humans and mice in a range of sites after epithelial damage but has presumably evolved primarily to cross an intact epithelium with trigeminal innervation. This could be oral, nasal, or ocular. Early murine studies used intranasal (i.n.) inoculation to reproduce human encephalitis, as the predominantly limbic distribution of HSV-1 lesions had suggested olfactory spread (14, 15). However, murine encephalitis affects mainly the brainstem (16, 17) and is largely limited to juveniles (18), whereas human disease is more common in adults; indeed, its poor correlation in age distribution with primary infection suggests that human encephalitis often results from viral reactivation rather than primary infection (5). Thus, recent analysis has focused mainly on acute and long-term infections of the TG, delivering virus to this site by scarification (19).

Recent insight into herpesvirus host entry has come from analysis of a murid gammaherpesvirus, MuHV-4. Like HSV-1, gammaherpesviruses are thought to infect orally. However, oral MuHV-4 was noninfectious (20), whereas i.n. virions efficiently infected the olfactory neuroepithelium (21). The molecular explanation was that only this epithelium expresses apically the heparan sulfate (HS) on which MuHV-4 cell binding and host entry (22) depend. This result suggested that other HS-binding herpesviruses, such as HSV-1 (23, 24), might also infect the neuroepithelium. Understanding MuHV-4 entry depended on reproducing so far as possible the likely conditions of natural virus uptake—small virus volumes and no anesthesia—and using reporter constructs to reveal asymptomatic infection. Here, we applied the same approach to HSV-1. Ocular infection works poorly without scarification (25), so we compared oral and nasal inoculations. Nasal HSV-1 was much more infectious. Like MuHV-4, it targeted the olfactory neuroepithelium. It did not cause encephalitis but rather reached the TG before reemerging in florid lesions of the facial skin. Thus, i.n. HSV-1 established a functional TG infection without requiring scarification or causing severe disease.

## MATERIALS AND METHODS

### Mice.

BALB/c, C57BL/6 (Harlan OLAC), and ROSA26R mice (26) were infected at age 6 to 8 weeks (adults) or 1 to 2 weeks (pups). For adult i.n. infections, virus (10^6^ PFU in 5 μl unless stated otherwise) was pipetted onto the nares of mice held prone under light restraint without anesthesia and was spontaneously inhaled. Oral infections followed a similar scheme, but the mice were held supine and virus was pipetted into the mouth. For infant infections, HSV-1 (10^6^ PFU) was pipetted onto the nares in 2 μl and spontaneously inhaled. For lung infections, mice were anesthetized by isoflurane inhalation and given virus i.n. in 30 μl. For whisker pad infections, mice were anesthetized with isoflurane, a drop of virus (10^6^ PFU) was applied to the whisker pad, and 4 scratches were made through the drop with a 27-gauge needle. For luciferase imaging, mice were injected intraperitoneally with luciferin (2 mg/mouse), anesthetized with isoflurane, and imaged for light emission with a charge-coupled-device (CCD) camera (Caliper Life Sciences). Luciferase images were analyzed with Living Image software (Caliper Life Sciences). All animal experiments were approved by the University of Cambridge ethical review board and by the United Kingdom Home Office under the 1986 Animal (Scientific Procedures) Act as Project License 80/2538.

### Cells and viruses.

BHK-21 cells (American Type Culture Collection CCL-10) were propagated in Dulbecco's modified Eagle's medium, supplemented with 2 mM glutamine, 100 U/ml penicillin, 100 mg/ml streptomycin, and 10% fetal calf serum (PAA laboratories) (complete medium). All viruses were derived from HSV-1 strain SC16, a relatively virulent clinical isolate (18) that has not been extensively passaged *in vitro*. Virus stocks were grown and titers were determined on BHK-21 cells (27). Virus was recovered from infected cells plus supernatant by ultracentrifugation (38,000 × *g*, 90 min) and sonicated to break down cellular debris. SC16 derivatives with a human cytomegalovirus (HCMV) IE1 promoter transcribing enhanced green fluorescent protein (eGFP) (HSV-GFP) (27) or Cre recombinase (HSV-Cre) (28) from the Us5 locus have been described, as has eGFP-expressing MuHV-4 (29). To make luciferase-expressing HSV-1 (HSV-LUC), the Photinus pyralis luciferase coding sequence plus an upstream HCMV IE1 promoter and a downstream polyadenylation (pA) site were subcloned from pGL4.10 (Promega Corporation) into pHD5, thereby inserting the expression cassette at HSV-1 nucleotide 137945 in Us5 (27). Linearized plasmid was cotransfected with HSV-1 SC16 genomic DNA into BHK-21 cells using Fugene-6 (Roche Diagnostics Ltd.). Luciferase-positive viruses were identified by scintillation counting (20), purified by limiting dilution cloning, and confirmed as recombinant by restriction enzyme mapping of viral DNA.

### Virus assays.

Plaque assays were performed by culturing virus dilutions with BHK-21 cell monolayers for 2 h and then overlaying with complete medium plus 0.3% carboxymethylcellulose and culturing at 37°C. After 48 h, the monolayers were fixed in 4% formaldehyde and stained with 0.1% toluidine blue. Plaques were counted under ×30 microscopy. To measure preformed infectious virus, tissues (noses, lungs, skin) were freeze-thawed and then homogenized (Omni tissue grinder). To measure both preformed infectious and reactivatable virus, tissues (TG, olfactory bulbs [OB]) were disrupted by pipetting and then incubated (37°C, 30 min) with Liberase TL (2 Wünsch units [WU]/ml) and DNase I (0.2 mg/ml) (Roche Diagnostics Ltd.). The released cells were then plated on BHK-21 cell monolayers and cultured for 48 h to allow plaque formation. To count plaques, we fixed and stained samples as described above.

### Immunohistochemistry.

The nasal epithelium was removed postmortem as a block of tissue bounded by the cartilaginous tip of the nose anteriorly, the orbits posteriorly, the zygomatic arches laterally, the palate ventrally, and the nasal bones dorsally. Samples were fixed in phosphate-buffered saline (PBS)–2% formaldehyde (4°C, 24 h) and then decalcified by gentle agitation in 150 mM NaCl-50 mM TrisCl (pH 7.2)-270 mM EDTA for 2 weeks at 23°C, and the solution was changed every 2 to 3 days. Decalcified samples were washed 3 times in PBS, dehydrated in 70% ethanol, and paraffin embedded. Skin was cut into strips and then fixed in PBS-2% formaldehyde (4°C, 24 h) before dehydration in 70% ethanol and embedding in paraffin. TG were removed postmortem, fixed in PBS-2% formaldehyde (4°C, 24 h), and decalcified for 1 week to remove residual bone fragments before dehydration in ethanol and embedding in paraffin. Sections (7 μm) were cut from paraffin-embedded tissues with a microtome and then dewaxed in xylene and hydrated in graded ethanol solutions for immunostaining. Endogenous peroxidase activity was quenched in PBS-3% H_2_O_2_ for 10 min. Sections were blocked with the avidin/biotin blocking kit (Vector Laboratories) and in PBS-2% bovine serum albumin (BSA)-2% normal serum from the species providing the secondary antibody (1 h, 23°C). HSV-1 antigens were detected with a rabbit polyclonal antibody (pAb) and eGFP with rabbit anti-eGFP pAb (Abcam). After being stained (18 h, 23°C), the sections were washed 3 times in PBS, incubated (30 min, 23°C) with biotinylated goat anti-rabbit IgG pAb (Vector Laboratories), washed 3 times in PBS, incubated with the Vectastain Elite ABC peroxidase system, washed 3 times in PBS, and developed with ImmPACT DAB (3,3′diaminobenzidine) substrate (Vector Laboratories). The sections were then counterstained with Mayer's hemalum (Merck), dehydrated, and mounted in DPX (BDH).

### Immunofluorescence.

Samples were fixed in 1% formaldehyde-10 mM sodium periodate-75 mM l-lysine (4°C, 24 h), equilibrated in 30% sucrose (4°C, 18 h), and then frozen in OCT and sectioned (7 μm) on a cryostat. Sections were air dried (2 h, 23°C) and blocked with 2% goat serum-2% BSA-PBS (1 h, 23°C). Primary antibody incubations were as described for immunohistochemistry. We additionally detected α-tubulin with a rat monoclonal antibody (MAb) (Serotec), nectin-1 with a rabbit pAb (Santa Cruz Biotech), ZO-1 with a rabbit pAb (Invitrogen), and CD68 with a rat MAb (BioLegend). After incubation, sections were washed 3 times in PBS and then incubated (1 h, 23°C) with Alexa Fluor 568- or 633-conjugated goat anti-rat IgG pAb and Alexa Fluor 488- or 568-conjugated goat anti-rabbit IgG pAb (Invitrogen). After 3 further washes in PBS, the sections were mounted in Prolong Gold-DAPI (4′,6-diamidino-2-phenylindole) (Invitrogen). Fluorescence was visualized using a Leica TCS SP2 confocal microscope and analyzed with ImageJ.

### β-Galactosidase assay.

TG and OB were fixed in 4% formaldehyde (1 h, 4°C) and then washed 3 times in PBS and incubated in PBS with 0.01% sodium deoxycholate, 0.02% NP-40, 2 mM MgCl_2_, 4.5 mM potassium ferrocyanide, 4.5 mM potassium ferricyanide, and 0.4 mg/ml X-Gal (5-bromo-4-chloro-3-indolyl-β-d-galactopyranoside) (18 h, 37°C). TG and OB were then rinsed in PBS and squashed between two coverslips to reveal any X-Gal staining, and blue cells were counted under ×40 to ×100 microscopy.

### Flow cytometry.

Cells exposed to eGFP-expressing viruses were trypsinized, washed in PBS, and analyzed for green channel fluorescence on a FACSCalibur using CellQuest software (BD Biosciences).

### ELISA.

Maxisorp enzyme-linked immunosorbent (ELISA) plates (Nalge Nunc) were coated (18 h, 4°C) with 0.05% Triton X-100-disrupted HSV-1 virions. Plates were washed 3 times with PBS-0.1% Tween 20, blocked with PBS-0.1% Tween 20-1% BSA, incubated with serum dilutions (1 h, room temperature), washed 4 times in PBS-0.1% Tween 20, incubated with alkaline phosphatase-conjugated goat anti-mouse IgG pAb, washed 5 times in PBS-0.1% Tween 20, and developed with nitrophenylphosphate substrate (Sigma Chemical Co.). The reaction was terminated with NaOH, and the absorbance was read at 405 nm (Bio-Rad Benchmark ELISA plate reader).

## RESULTS

### HSV-1 infects more efficiently i.n. than p.o.

**Fig 1:**
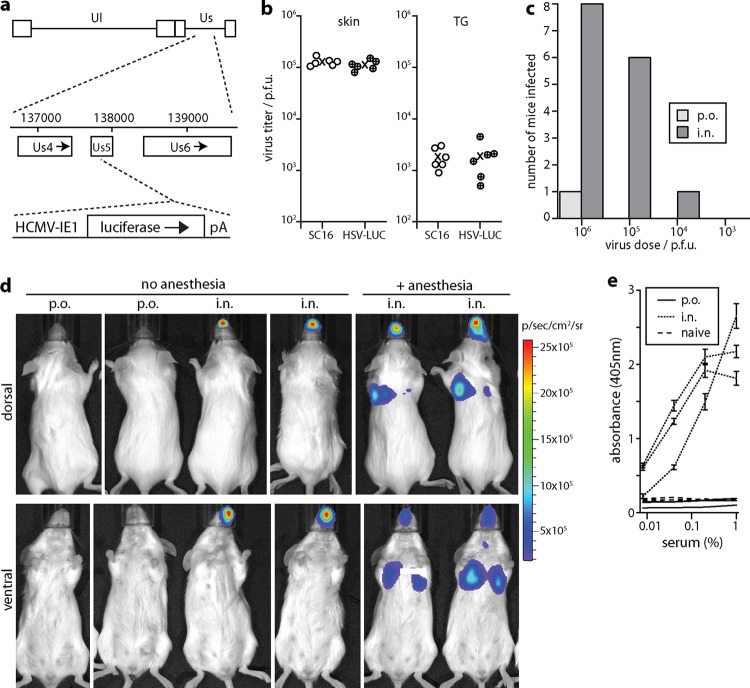
Comparison of HSV-1 inoculation routes. (a) Schematic diagram showing luciferase expression cassette insertion into the HSV-1 SC16 Us5 locus to generate HSV-LUC. (b) Mice were infected in the whisker pad with either HSV-LUC or the parental virus SC16. Five days later, virus titers were determined by plaque assay from homogenates of either whisker pad skin or TG. Circles show individual mice, and exes show means. There was no significant difference in titers between viruses in either site (*P* > 0.4 by Student's 2-tailed *t* test). (c) BALB/c mice (6 to 8 weeks old, 8 per group) were given various HSV-LUC doses either orally (p.o.) or intranasally (i.n.) in 5-μl doses without anesthesia and then monitored daily for luciferase expression over a week. They were considered infected if they showed light emission above the background of an uninfected mouse (10^5^ photons/s/cm^2^/sr). Luciferase-negative mice were confirmed as uninfected by ELISA for HSV-1-specific serum IgG (see panel e). (d) Mice were inoculated with HSV-LUC (10^6^ PFU), i.n. or p.o. in 5-μl doses, as described for panel c, or i.n. in 30-μl doses under general anesthesia. Two days later, luciferase expression was imaged by intraperitoneal (i.p.) luciferin injection and CCD camera scanning. Representative images are shown for 2 mice per group. Light flux for p.o. inoculations was undetectable (<10^5^ photons/s/cm^2^). (e) One month after p.o. or i.n. HSV-LUC inoculations, as described for panel c (5 μl, 10^6^ PFU), mice were tested by ELISA for HSV-1-specific serum IgG. Three representative mice per group are shown plus age-matched, naive controls. Each point shows the mean ± standard deviation (SD) from triplicate samples.

Viral luciferase expression allows infection tracking independent of clinical signs or assumptions about its timing and distribution. Luciferase-expressing HSV-1 KOS has been tracked after scarification (30). However, the KOS strain is attenuated (31). Less invasive inoculations demand greater viral replicative fitness. A transgenic luciferase reporter mouse can reveal lytic infection by any HSV-1 strain but has limited sensitivity (32). Therefore, instead we expressed luciferase from an HCMV IE1 promoter in the Us5 locus of a relatively virulent strain, SC16 (18) (Fig. 1a). Us5 inhibits apoptosis (33), but its disruption has little effect on HSV-1 replication (34), and the recombinant virus (HSV-LUC) infected mice comparably to its SC16 parent after whisker pad scarification (Fig. 1b).

We compared 5 μl p.o. and i.n. inoculations of alert, adult mice (Fig. 1c). Intranasal administration of HSV-1 was ∼100-fold more infectious than oral administration. Thus, the nasopharynx was a more efficient entry route than the oropharynx. Figure 1d shows a typical experiment, with no infection by p.o. virus, nose infection by i.n. virus in alert mice, and nose plus lung infection by i.n. virus in anesthetized mice. Noninfection of luciferase-negative mice was confirmed by ELISA for HSV-1-specific serum IgG (Fig. 1e). HSV-1-specific IgG was detectable in sera of i.n. inoculated mice at a 1/270,000 dilution, whereas that in sera from p.o.-inoculated mice was undetectable at a 1/100 dilution. Thus, there was no sign of a significant infection occurring in luciferase-negative mice.

Not only was infection rare after p.o. inoculation, but we saw no oral infection in >40 mice given i.n. HSV-LUC, despite much of a 5-μl i.n. inoculum being swallowed (P.G. Stevenson, unpublished data). When oral infection occurred after p.o. inoculation, it affected the gums or tongue but was inconsistent in site. Thus, it possibly reflected epithelial damage during inoculation, particularly as alert mice often bit the inoculating pipette tip. Epithelial damage during inoculation could also explain reports of infection by gavage (35).

Alert, adult BALB/c mice given 10^7^ PFU HSV-LUC i.n. showed ruffled fur and 10 to 20% weight loss but recovered (0/6 deaths). A total of 10^6^ PFU caused little obvious illness, although it was lethal to 1/5 juvenile (3-week-old) BALB/c mice. The SC16 wild type (10^6^ PFU) was similarly lethal to 0/5 adults and 3/5 juveniles. Thus, in adult mice, i.n. HSV-1 did not inevitably spread to the brain to cause encephalitis. In contrast, HSV-LUC delivered i.n. under anesthesia was lethal to 6/6 adult mice given 10^7^ PFU and 3/6 given 10^6^ PFU. Thus, when HSV-1 is given i.n. under anesthesia, lung infection may be a significant cause of disease.

### Intranasal infection progresses from the nose to trigeminal ganglia to the skin.

**Fig 2:**
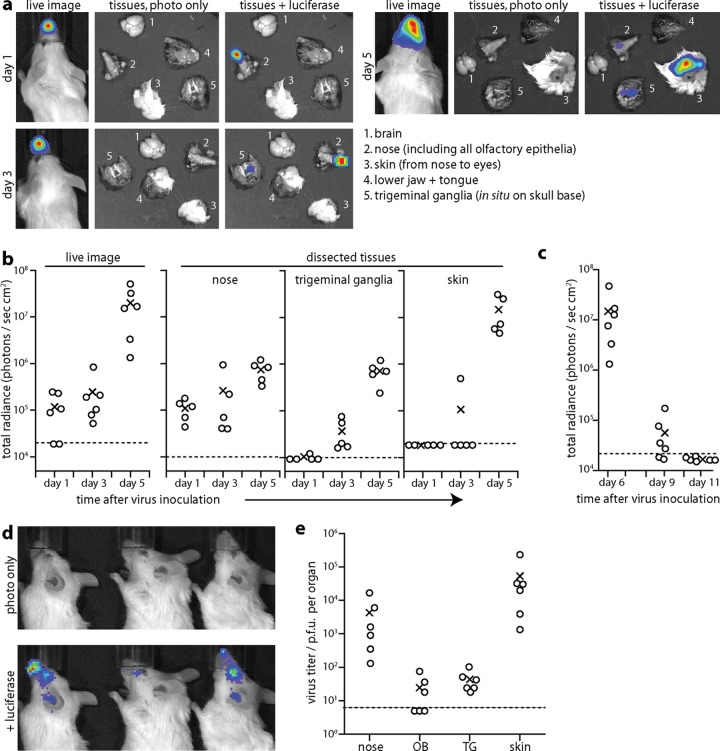
HSV-1 spread monitored by luciferase expression. (a) Six- to eight-week-old BALB/c mice were given HSV-LUC i.n. without anesthesia (10^6^ PFU) and imaged for light emission 1, 3, or 5 days later, first live (left panels) and then after dissection to identify the sources of the live image signals (right panels, shown with or without luciferase overlays). Images are shown for a representative mouse. (b) Mice were infected and analyzed as described for panel a, and signals were quantitated for 5 or 6 mice per group. Circles show individuals, and exes show means. Dashed lines show lower detection limits. These correspond to the photons detectable from an equivalent uninfected sample and hence are specific to each tissue, with white fur having a higher background than dissected noses or TG. The region of interest for live images extended from nose to ears and so covered all areas showing positive luciferase signals. (c) Mice were infected as described for panel a and imaged during the resolution of infection. (d) Representative images at day 9 after i.n. HSV-LUC inoculation show how infection has spread from the nose to the facial skin and eye, consistent with reactivation from the TG. (e) Mice were infected as described for panel a, and 6 days later titers were determined for infectious virus by plaque assay (nose, skin) or for infectious and reactivatable virus by explant (OB, olfactory bulbs; TG, trigeminal ganglia). Circles show individuals, and exes show means. Dashed lines show lower detection limits. Thus, virus was recovered from 6/6 TG and 3/6 OB.

Intranasal infection appeared to spread locally over the snout from day 1 to day 5 (Fig. 2a). However, postmortem dissections established that the day 1 and day 5 luciferase signals had very different sources: day 1 signals were confined to the nose, whereas day 5 signals were >95% from the skin (Fig. 2b). The skin signal was variable in site but was always contained within the region bounded by the ears posterolaterally and nose anteriorly. In ∼30% of mice, day 5 infection also involved the eye. At day 3 postinoculation, TG of i.n.-infected mice were luciferase positive, whereas the skin was rarely so. Thus, the order of infection was nose, then TG, and then skin (Fig. 2b). At day 5, 1/12 mice showed an OB signal, 3/12 mice showed a weak brainstem signal, and 0/12 mice showed a cerebral cortex signal. Thus, the brain was largely luciferase negative. All luciferase signals had decreased markedly by day 9 and were absent by day 11 (Fig. 2c). As at day 5, the day 9 signals (Fig. 2d) involved the dermatomes of the (ophthalmic and maxillary) TG branches that innervate the nose. Plaque assays at day 6 postinoculation (Fig. 2e) recovered high titers of infectious virus from skin and noses. Lower and more variable virus titers were recovered by explant of TG (6/6 mice) and OB (3/6 mice).

**Fig 3:**
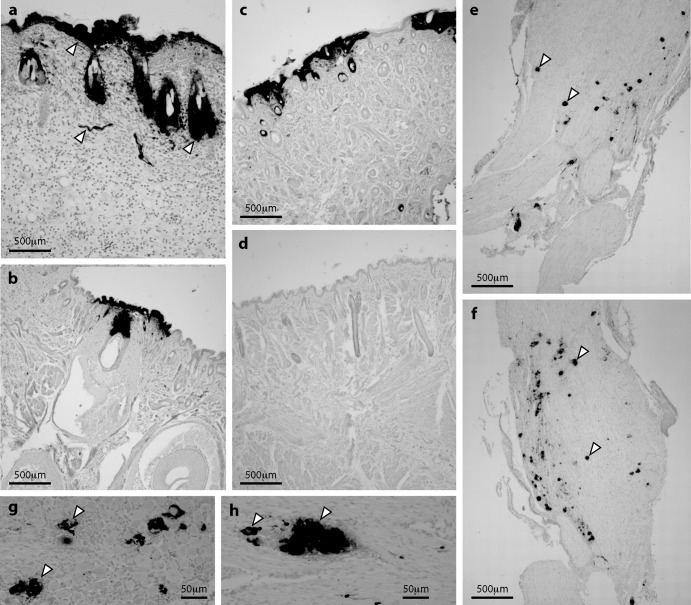
HSV-1 antigen expression in skin and TG. (a to c) Six- to eight-week-old BALB/c mice were infected i.n. with HSV-LUC (10^6^ PFU). Five days later, luciferase-positive skin sections were stained for HSV-1 antigens with a polyclonal immune serum (dark staining, arrows in panel a show examples) and counterstained with Mayer's hemalum. The images are representative of 6 mice examined. (d) Uninfected skin, stained and counterstained as described for panels a to c. (e and f) TG of mice infected as described for panel a were stained for HSV-1 antigens. Representative images are shown. Arrows show example positive cells. (g and h) Patches of HSV-1 antigen staining in TG of mice infected as described for panel a. Arrows show examples.

Immunohistochemical staining showed strong HSV-1 antigen expression in the epidermis and hair follicles of luciferase-positive skin at day 5 (Fig. 3a to d). The lesions were patchy, suggesting that virus had reached here not by contiguous spread from the nasal mucosa but by reemergence from TG neurons, as occurs after corneal scarification (36, 37). Lytic antigen expression was evident in acutely infected TG (Fig. 3e to f), and groups of adjacent infected cells (Fig. 3g to h) suggested infection spread between neurons, possibly within the TG or between nerve terminals in the brainstem. This would explain how i.n. virus could pass from the nose to the skin via the TG. The abundance of luciferase expression, infectious virus, and viral antigen staining in skin implied that emerging virus was also amplified by local lytic spread.

### Cre-expressing HSV-1 in reporter mice confirms trigeminal infection by the i.n. route.

**Fig 4:**
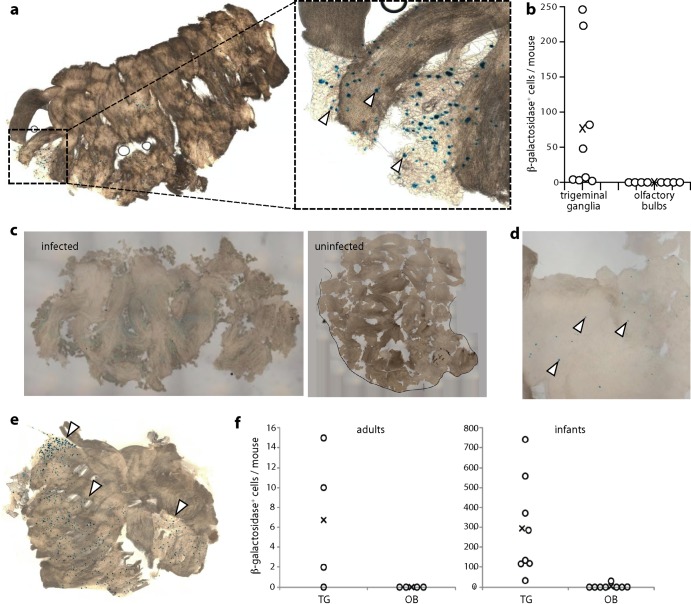
Floxed reporter gene activation by viral Cre recombinase. (a) ROSA26R mice (6 to 8 weeks old), which have a floxed β-galactosidase expression cassette activated by Cre recombinase, were infected i.n. (10^6^ PFU) with HSV-1 expressing Cre from an HCMV IE1 promoter (HSV-Cre). Ten days later, TG and OB were removed, fixed, incubated with X-Gal, and examined for β-galactosidase-positive cells (blue spots). A representative infected TG is shown. The arrows in the zoomed image show example positive cells. Uninfected ROSA26R mice and infected, nontransgenic C57BL/6 controls showed no blue spots. (b) Mice were infected and analyzed as described for panel a, and blue spots were counted for TG and OB. Circles show individual mice, and exes show means. All TG contained blue cells, although the number per mouse was variable. No OB contained blue cells. (c) An OB of a strongly infected ROSA26R mouse inoculated i.n. 10 days earlier with HSV-Cre and developed with X-Gal showed a faint blue wash, but no blue cells. The blue wash possibly reflected enzyme or converted substrate leaking from the axons of β-galactosidase-positive primary olfactory neurons. An uninfected OB is shown for comparison. (d) At 20 days after i.n. HSV-Cre inoculation of a 1-week-old mouse, an OB shows a few, scattered blue cells (arrows). Magnification is ×5 relative to that of panel c. (e) A TG from the same mouse shows large numbers of blue-staining cells (arrows). (f) Eight-week-old (adult) or 1-week-old (infant) ROSA26R mice were infected with HSV-Cre and 20 days later analyzed for β-galactosidase expression in TG and OB. Circles show individual mice, and exes show means. Three out of four adult and 8/8 infant TG and 0/4 adult and 1/8 infant OB contained blue cells. In this experiment, adult TG blue cell counts were at the lower end of the range seen in panel b, and infant counts were 50-fold higher.

To explore further HSV-1 infection spread from the nose, we used a recently described approach (28) in which HCMV IE1 promoter-driven viral Cre expression permanently marks infected cells of mice transgenic for a floxed reporter gene. Thus, infected cells can be identified regardless of whether viral gene expression is maintained. We gave HSV-Cre i.n. to ROSA26R mice (26), in which *loxP* recombination activates β-galactosidase production from a widely expressed promoter, and 10 days later identified Cre-switched cells by incubating fixed tissues with X-Gal (Fig. 4a and b).

The TG of individual infected mice varied widely in β-galactosidase-positive cell numbers, but all (8/8) showed some evidence of infection, whereas dissociated OB showed none (0/8). One OB of a heavily infected mouse showed weak, diffuse staining (Fig. 4c) that may have reflected β-galactosidase leaking from the axons of infected primary olfactory neurons, as these are sheared at the cribriform plate when the OB are removed. This would also explain the recovery of some infectious virus from OB samples (Fig. 2e). We saw no evidence of infection passing to cells within the OB.

### Intranasal infection of infant mice similarly shows TG infection.

Most HSV-1 infections occur in early childhood, and the outcome of infection can be age dependent (18). We therefore also compared i.n. HSV-Cre infections of adult (8-week-old) and infant (1-week-old) ROSA26R mice (Fig. 4d and f). Infant infections were more extensive than those of adults (TG contained 4- to 40-fold more β-galactosidase-positive cells). Nevertheless, infant OB contained at most a few, scattered β-galactosidase-positive cells (Fig. 4d), in contrast to abundant TG infection (Fig. 4e). At 20 days postinoculation, incubation with X-Gal revealed β-galactosidase-positive cells in 3/4 TG and 0/4 OB of ROSA26R adults and 8/8 TG and 1/8 OB of infants (Fig. 4f). Thus, in both infants and adults, i.n. HSV-1 showed a strong predominance of TG over OB involvement. Immunostaining also showed much more TG and brainstem infection than OB infection in infant mice (data not shown), consistent with previous reports (16, 17, 38, 39).

### Intranasally administered HSV-1 targets mainly the olfactory neuroepithelium.

**Fig 5:**
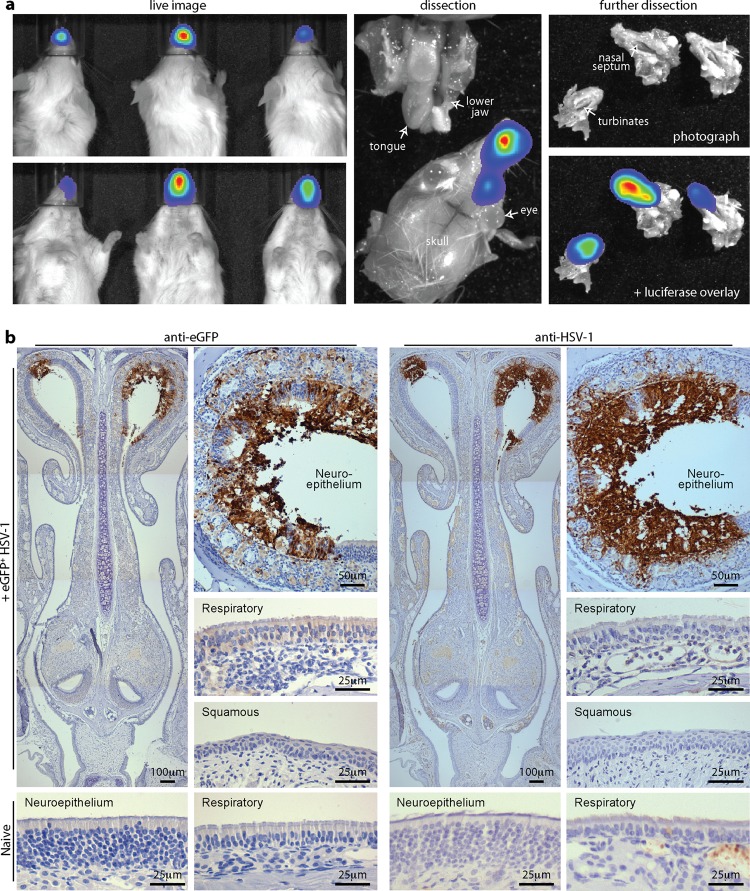
HSV-1 infection of the adult olfactory neuroepithelium. (a) Six- to eight-week-old BALB/c mice were inoculated i.n. with HSV-LUC (10^6^ PFU in 5 μl). One day later, infection was imaged by luciferin injection and CCD camera scanning. Dissections localized luciferase signals to the nasal septum and turbinates, with none from the oral cavity. Equivalent results were obtained with >20 mice. (b) Six- to eight-week-old BALB/c mice were infected i.n. with HSV-eGFP (10^6^ PFU in 5 μl). Three days later, dissected tissues were processed for immunohistochemistry. The images, representative of 12 mice examined, show staining (brown) for viral eGFP (left-hand set of images) and HSV-1 lytic antigens (right-hand set of images). Sections were counterstained with Mayer's hemalum. The insets show higher-magnification images, including representative regions of respiratory and squamous epithelium, plus neuroepithelial and respiratory epithelial samples of uninfected controls.

Intranasally administered MuHV-4 targets the olfactory neuroepithelium, which lines the upper nasal septum and turbinates, whereas influenza virus targets the more anterior respiratory epithelium (21). Postmortem dissections of adult mice given i.n. HSV-LUC localized day 3 luciferase signals to the nasal septum and turbinates (Fig. 5a), with no oral signal, a pattern very similar to that seen with MuHV-4 (21). To track infection more precisely, we infected adult mice with HSV-GFP (27) and identified infected cells by immunostaining (Fig. 5b). This showed both eGFP and virion antigens in the neuroepithelium.

**Fig 6:**
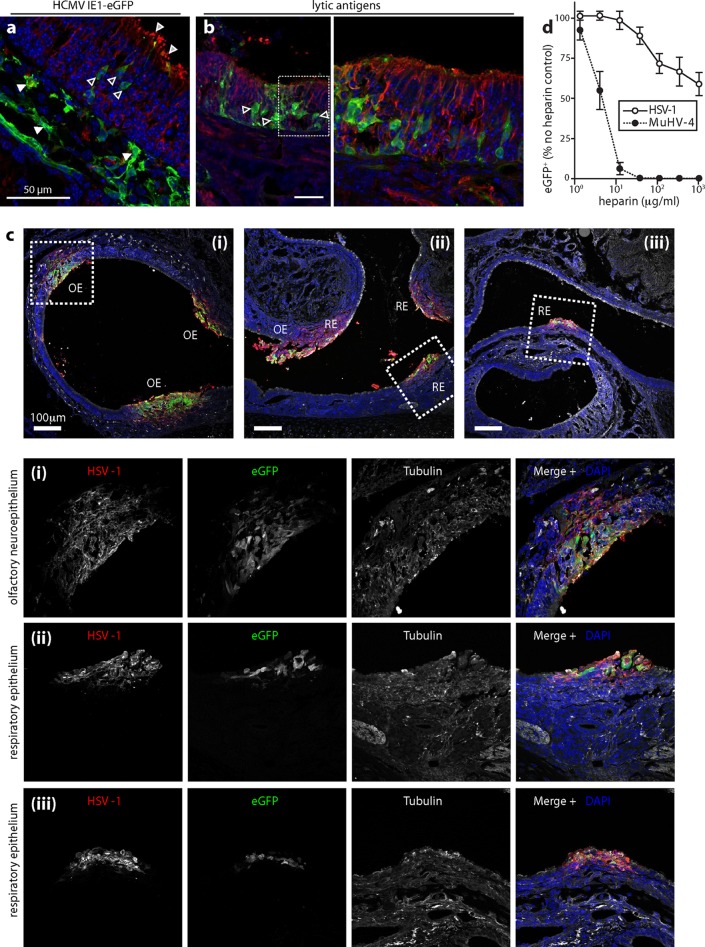
HSV-1 infection of the infant olfactory neuroepithelium. (a) One- to two-week-old mice were infected i.n. with HSV-eGFP (10^6^ PFU in 2 μl). Three days later, nose sections were stained for eGFP (green) and α-tubulin (red). Nuclei were stained with DAPI (blue). Light-gray-filled arrows show α-tubulin staining on the apical neuronal cilia; dark-gray-filled arrows show eGFP-positive neurons; white arrows show subepithelial eGFP-positive cells. (b) Mice were infected as described for panel a. Three days later, nose sections were stained for viral antigens (green) and α-tubulin (red). Nuclei were stained with DAPI (blue). The right-hand image shows the boxed region of the corresponding left-hand image at higher magnification. The gray-filled arrows show examples of eGFP-positive neuroepithelial cells. (c) One- to two-week-old BALB/c mice were infected i.n. with HSV-eGFP (10^6^ PFU in 2 μl). One day later, noses were stained for eGFP (green), HSV-1 virion antigens (red), and α-tubulin (white). Nuclei were stained with DAPI (blue). Panels i, ii, and iii show different regions of nasal epithelia, either olfactory (OE) or respiratory (RE). Squamous epithelium showed no staining. The boxed regions are shown at higher magnification with individual channels below. (d) HSV-GFP and eGFP-expressing MuHV-4 were incubated with various concentrations of heparin (1 h, 37°C) and then added to BHK-21 cells (0.5 PFU/cell, 37°C). Eighteen hours later, cells were scored as eGFP positive or negative by flow cytometry. Each point shows means ± SD from 3 experiments, with 20,000 cells analyzed for each point in each experiment. The inhibition of HSV-1 infection by heparin was statistically significant at all doses of >10 μg/ml (*P* < 10^−4^ by chi-square test), but the inhibition of MuHV-4 infection was always significantly greater (*P* < 10^−6^ by chi-square test).

Infected infant mice (Fig. 6) similarly showed neuroepithelial eGFP (Fig. 6a) and lytic antigen expression (Fig. 6b). Some mice (∼20%) also showed patches of respiratory epithelial infection (Fig. 6c). Thus, the neuroepithelial infection preference of HSV-1, while marked, was less exclusive than that of MuHV-4. Neuroepithelial binding and infection by MuHV-4 correlate with its capacity and requirement for HS binding (21). Although HSV-1 is well known to bind HS, the importance of this interaction for infection has been assessed mainly by adding soluble heparin to plaque assays. Plaque formation requires multiple rounds of infection, so heparin can inhibit infection multiple times in a single assay. We used HSV-GFP to assay heparin inhibition of a single-cycle infection and compared it with eGFP-expressing MuHV-4. Although HSV-1 infection was inhibited by heparin, the inhibition was much less marked than for MuHV-4. This was consistent with HSV-1 and MuHV-4, both encoding HS binding glycoproteins (24, 40, 41), but only MuHV-4 also encodes a glycoprotein (gp150) that inhibits HS-independent cell binding (42).

**Fig 7:**
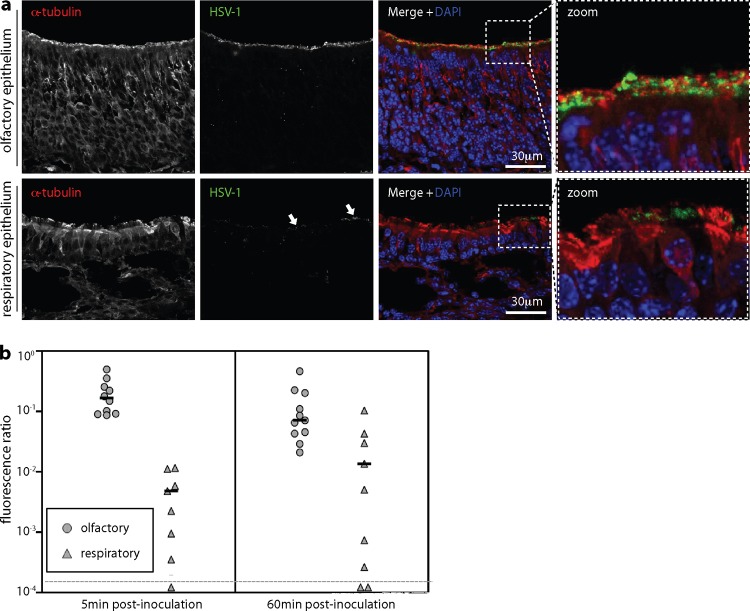
Epithelial binding by i.n. HSV-1. (a) Mice were inoculated i.n. with HSV-1 (10^6^ PFU in 5 μl) or not (naive). Five minutes later, noses were flushed with PBS and sections were stained for viral antigens with a polyclonal immune serum and for α-tubulin to reveal the apical extents of the neuronal and respiratory epithelia. Nuclei were counterstained with DAPI. Representative sections are shown. The right-hand panels show the boxed regions of the composite images at higher magnification. Arrows show the minor, patchy staining of respiratory epithelium. (b) Mice were inoculated with HSV-1 and analyzed for virion binding as described for panel a. Binding was quantitated by counting HSV-1-positive pixels over a fixed area of apical epithelium and then normalizing by the α-tubulin signal of the same area. Each circle or triangle shows the result for 3 sections of 1 mouse. The horizontal bars show medians. Student's unpaired, 2-tailed *t* test showed that virion binding to the olfactory neuroepithelium was significantly greater than binding to the respiratory epithelium at both 5 min (*P* < 0.002) and 60 min (*P* < 0.05). Equivalent data were obtained in 1 further experiment.

**Fig 8:**
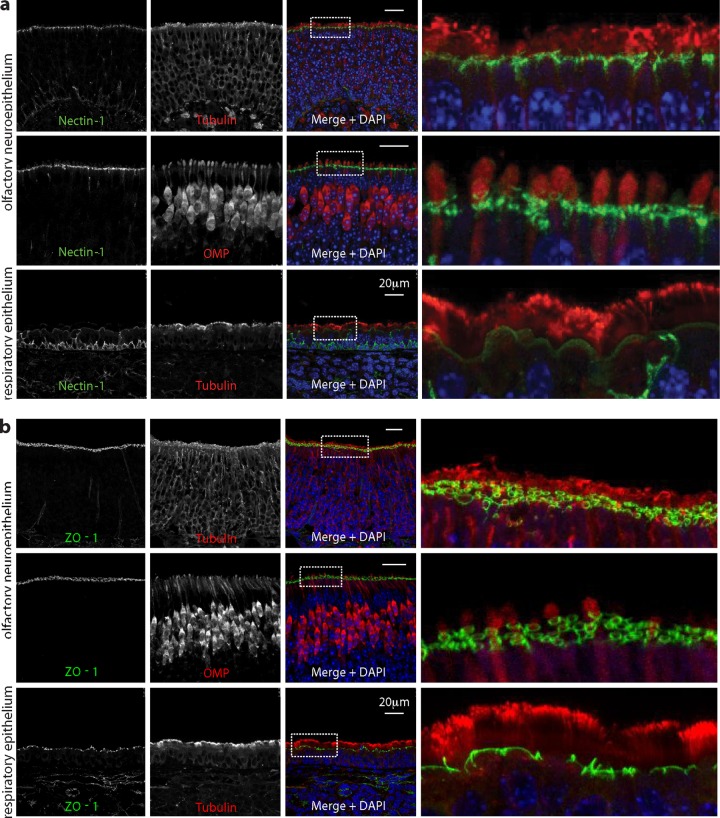
Nectin-1 expression by nasal epithelia. (a) Nasal epithelia of naive mice were stained for the HSV-1 gD receptor nectin-1 (green) and costained for α-tubulin or olfactory marker protein (red). Nuclei were stained with DAPI (blue). Representative regions of neuroepithelium and respiratory epithelium are shown. The right-hand panels show the boxed regions at higher magnification. (b) Nasal epithelia of naive mice were stained for the tight junction component ZO-1 and costained for α-tubulin or olfactory marker protein (red). Nuclei were stained with DAPI (blue). Representative regions of neuroepithelium and respiratory epithelium are shown. The right-hand panels show the boxed regions at higher magnification. ZO-1 and nectin-1 showed similar distributions.

We next looked at virion binding (Fig. 7). After i.n. inoculation, some HSV-1 antigens bound to the respiratory epithelium (Fig. 7a), but quantitation consistently showed more binding to the neuroepithelium (Fig. 7b). Nectin-1 engagement by gD plays a prominent role in HSV-1 neuronal infection (43), and immunostaining (Fig. 8a) identified abundant nectin-1 expression just below the neuroepithelial cilia. Apical nectin-1 expression by the respiratory epithelium was less marked. Where the neuroepithelial cilia terminate, olfactory neurons form tight junctions with the adjacent sustentacular cells, as revealed by staining for the tight junction component ZO-1 (Fig. 8b). Thus, nectin-1 was abundantly expressed just below HS-positive neuronal cilia, suggesting that the neuroepithelial infection preference of HSV-1 may reflect the availability of nectin-1 as well as HS.

## DISCUSSION

The ready transmission of pseudorabies, varicella-zoster, and Marek's disease viruses (10, 11, 44) suggests respiratory infection. HSV-1 needs closer contact to transmit frequently. However, there is no obvious need to invoke a different entry route: lower transmission rates could reflect simply less virus shedding. HSV-1 infected mice much more efficiently i.n. than p.o. and targeted mainly the olfactory neuroepithelium to reach the TG. Thus, this surface provides access to the persistent reservoirs of both an alphaherpesvirus (HSV-1) and a gammaherpesvirus (MuHV-4). The respiratory spread of pseudorabiesvirus tells us that species other than mice readily inhale infectious saliva; viruses such as measles spread between humans in this way; and varicella-zoster virus spreads between humans by saliva or vesicle fluid inhalation. Thus, even small amounts of HSV-1 in saliva could plausibly achieve respiratory transmission by close contact.

The assumption of oral human HSV-1 entry has arisen mainly from infection often presenting with oral lesions. A lack of oral infection in mice does not refute this assumption: humans could express an oral HSV-1 receptor that mice lack, or extensive oral salivary exchange could promote human oral infection even if it is poorly efficient. However, interpreting clinical lesions is not straightforward. Herpesviruses enter via epithelia, establish a more systemic latent infection, and then reemerge via epithelia for host exit. Thus, epithelial lesions could reflect either host entry or exit. The presenting skin lesions of acute varicella-zoster virus infection are clearly not host entry. The tonsillitis of acute Epstein-Barr virus infection similarly occurs at least a month after virus acquisition (45), when viral shedding is at its peak; at an equivalent time after intranasal administration of MuHV-4, infection is mainly in the cervical lymph nodes and genital tract rather than the nose (46). And human cytomegalovirus presents with systemic symptoms and virus shedding from multiple sites. Thus, herpesvirus infections seem to present consistently with secondary lesions after asymptomatic host entry. The well-described phenomenon of zosteriform spread (8, 36, 37) corresponds to HSV-1 exiting acutely from sensory ganglia to cause more widespread lesions than its initial entry; the extensive neuronal connections of the TG provide an abundant scope for such spread; and 5 days after i.n. HSV-1 inoculation, lesions were largely in TG-innervated skin rather than the nose. Thus, the acute oral lesions of human HSV-1 infection need not necessarily reflect oral host entry.

Other, general considerations *vis-à-vis* oral entry are that HSV-1 virions are acid sensitive—most p.o.-transmitting viruses have evolved resistance to low pH because most saliva is rapidly swallowed—and that efficient oral virion capture is hard to reconcile with efficient salivary shedding, as carriers would tend to recapture their released virions. The opposing demands of virion capture and release are more readily reconciled if host entry and exit follow different routes. HS binding allows efficient virion uptake by HS-positive olfactory neuronal cilia and efficient virion release from oral epithelia that express only basolateral HS (33). We also saw strong neuroepithelial expression of the gD receptor nectin-1 at subcilial tight junctions. An important task now for designing interventions is to establish the quantitative importance of neuroepithelial HS and other receptors in i.n. host entry, for example, by using receptor knockout mice.

A common perception is that viruses readily spread from olfactory neurons to the brain and that i.n. HSV-1 inevitably spreads to the OB. This would make nasal entry unlikely, as human OB are rarely infected. However, adult murine OB involvement by i.n. HSV-1 was also rare. Others have found this for lytic infection (47), and the lack of a viral Cre recombinase footprint in floxed reporter gene mice showed that most i.n.-infected mice lack even latent OB infection. OB involvement seems to occur mainly in severe infections, for example, in juvenile mice, and even then later and less consistently than TG involvement (17, 38), suggesting that secondary virus spread, from meninges (48), bloodstream (39), or other brain regions, is more common than direct passage along olfactory neurons. Vomeronasal organ involvement (49) may similarly reflect secondary spread during severe infection, as our milder i.n. infections never showed it. Thus, despite the capacity of HSV-1 to move along neurons (50, 51), neuroepithelial infection does not routinely spread in this way to the OB. Indeed, although some viruses can travel along olfactory neurons to cause encephalitis (52, 53), outside experimental settings, such disease is rare.

In summary, human clinical lesions clearly identify oral HSV-1 exit, but oral entry has always been more speculative: only deliberate virus inoculation can determine entry sites with any certainty. Within the limits of murine infection, nasal virus entered much more efficiently than oral virus, with surprisingly close parallels between HSV-1 and MuHV-4. From the neuroepithelium, HSV-1 readily reached the TG and thence the TG-innervated nonnasal regions characteristically colonized in humans. Thus, the possibility of i.n. HSV-1 host entry in humans should be reassessed with the aim of improving infection control.
